# A GC-MS Protocol for Separating Endangered and Non-endangered *Pterocarpus* Wood Species

**DOI:** 10.3390/molecules24040799

**Published:** 2019-02-22

**Authors:** Maomao Zhang, Guangjie Zhao, Juan Guo, Bo Liu, Xiaomei Jiang, Yafang Yin

**Affiliations:** 1 Department of Wood Anatomy and Utilization, Research Institute of Wood Industry, Chinese Academy of Forestry, Beijing 100091, China; maomaozhang1@gmail.com (M.Z.); guojuanchina@126.com (J.G.); liubo@criwi.org.cn (B.L.); xiaomei@caf.ac.cn (X.J.); 2 College of Materials Science and Technology, Beijing Forestry University, Beijing 100083, China; zhaows@bjfu.edu.cn; 3 Wood Collections (WOODPEDIA), Chinese Academy of Forestry, Beijing 100091, China

**Keywords:** GC-MS, xylariums, orthogonal partial least squares-discriminant analysis (OPLS-DA), species level, wood identification, extract

## Abstract

*Pterocarpus santalinus* and *Pterocarpus tincorius* are commonly used traded timber species of the genus *Pterocarpus*. *P. santalinus* has been listed in Appendix II of the Convention on International Trade in Endangered Species of Wild Fauna and Flora (CITES). As a non-CITES species, *P. tincorius* is also indiscriminately labeled as *P. santalinus* due to the similar macroscopic and microscopic features with *P. santalinus.* In order to understand the molecular discrimination between these easily confused species, xylarium heartwoods of these two species were extracted by three different kinds of solvents and analyzed using gas chromatography–mass spectrometry (GC-MS). Multivariate analyses were also applied for the selection of marker compounds that are distinctive between *P. santalinus* and *P. tincorius*. A total of twenty volatile compounds were detected and tentatively identified in three kinds of extracts, and these compounds included alcohols, stilbenoids, esters, aromatic hydrocarbons, ketones, miscellaneous, phenols, and flavonoids. GC-MS analyses also revealed that extraction solvents including ethanol and water (EW), ethyl acetate (EA), and benzene–ethanol (BE) gave the best chemotaxonomical discrimination in the chemical components and relative contents of the two *Pterocarpus* species. After chemometric analyses, EW displayed higher predictive accuracy (100%) than those of EA extract (83.33%) and BE extract (83.33%). Furthermore, spathulenol (17.58 min) and pterostilbene (23.65 min) were elucidated as the critical compounds for the separation of the EW extracts of *P. santalinus* and *P. tinctorius*. Thus, a protocol of GC-MS and multivariate analyses was developed to use for successfully distinguishing *P. santalinus* from *P. tinctorius*.

Academic Editor: Claire Turner

## 1. Introduction

Illegal logging seriously affects the global forest resources, causing forest destruction, the loss of biodiversity, climate change, and environmental deterioration [1,2]. The seizing of illegal wood products and the prosecuting of illegal logging crimes are of importance for the restriction of both illegal logging and associated trade [3]. Therefore, being able to identify timber to a level of certainty acceptable for admission to a court of law plays a critical role in the law enforcement for the forest protection [4]. 

The *Pterocarpus* spp. belong to the Leguminosae family, with approximately 35 species worldwide, which are widely distributed throughout the tropics of Africa, the Indo-Malayan region and North and South America [5]. The timber of *Pterocarpus* is well-known and highly valued due to its beautiful appearance, wood properties, medicinal properties, and even valuable bioactive compounds [6]. The high value has led to an increase in illegal logging of *Pterocarpus*, which results in a threat to wild *Pterocarpus* populations. Among the *Pterocarpus* genus, *P. santalinus*, which is primarily distributed in south India, is the most valuable species [7]. It is also the most commonly used wood species in the Chinese traditional furniture. Since 1995, *P. santalinus* has been listed in the CITES Appendix II. Another Pterocarpus species, *P. tinctorius*, is primarily distributed in the east, central, and southwest of Africa [8]. Although *P. santalinus* wood is restricted for trade, its products, such as furniture, artwares, and collections, are still very popular in the market. As a non-CITES species, *P. tinctorius* has similar macroscopic and microscopic features with *P. santalinus*, which has led to the confusion between them [6]. *P. tinctorius* is often deliberately labeled as *P. santalinus* by lawbreakers for illegal profits. Thus, developing accurate wood identification technology for *P. santalinus* and *P. tinctorius* is significant for both natural resource protection and market monitoring.

Traditional wood identification, which is based on wood macroscopic and microscopic features, is not able to identify wood at the species level. Besides, it is time-consuming and seriously relies on the experience of anatomists [4,6,9]. During the last decade, several new technologies, i.e., the genetic method based on DNA analysis and chemistry method, have been explored to overcome the above-mentioned shortcomings. DNA barcoding has been proved as an effective tool for wood identification at the species level, while its application is limited by the difficulties of extraction and amplification as well as the lack of a reliable library [10,11,12]. Chemistry method is an accurate and convenient tool for species identification due to the different chemical composition among species. The chemistry methods mainly included near infrared (NIR) spectrum, mid-infrared (IR) spectrum and GC-MS. Chemical fingerprint, based on NIR spectrum, has been used to distinguish red oak wood and white oak wood [13], to identify *Swietenia* [14], and to differentiate *Cryptomeria japonica* [15]. Chemical fingerprint based on IR spectrum has been also reported in the distinction of four *Dalbergia* species through the differences among their ethanol–benzene extractives [16,17,18]. However, both NIR and IR can only reflect the difference derived from chemical group vibrations and cannot figure out diagnostic compounds, which are critical for wood species identification.

GC-MS possesses high separation efficiency and reliable identification towards compounds. It is a powerful methodology for the study of chemical components of wood extractives or small molecule compounds produced by the degradation of cellulose, hemicellulose, and lignin [19,20,21,22,23,24,25]. In recent years, many researchers have focus on the application of GC-MS in the wood identification of *Phoebe zhennan* and *Machilus pingii* [26], and the chemotaxonomical discrimination of three similar *Dalbergia* species [22]. However, little research has been conducted to identify two similar *Pterocarpus* species—*P. santalinus* and *P. tinctorius*—based on GC-MS. Thus, xylarium heartwood specimens of *P. santalinus* and *P. tinctorius* were collected from curated xylaria collections, and three candidate extraction solvents (ethyl acetate, benzene–ethyl alcohol, and 1:1 mixture of ethyl alcohol and water) were selected to (1) explore the feasibility of wood identification between *P. santalinus* and *P. tinctorius* using GC-MS technology and (2) provide a protocol of GC-MS and multivariate analyses for the wood identification.

## 2. Results and Discussion

### 2.1. GC-MS Analysis of Heartwood Extracts of P. santalinus and P. tinctorius

**Figure 1:**
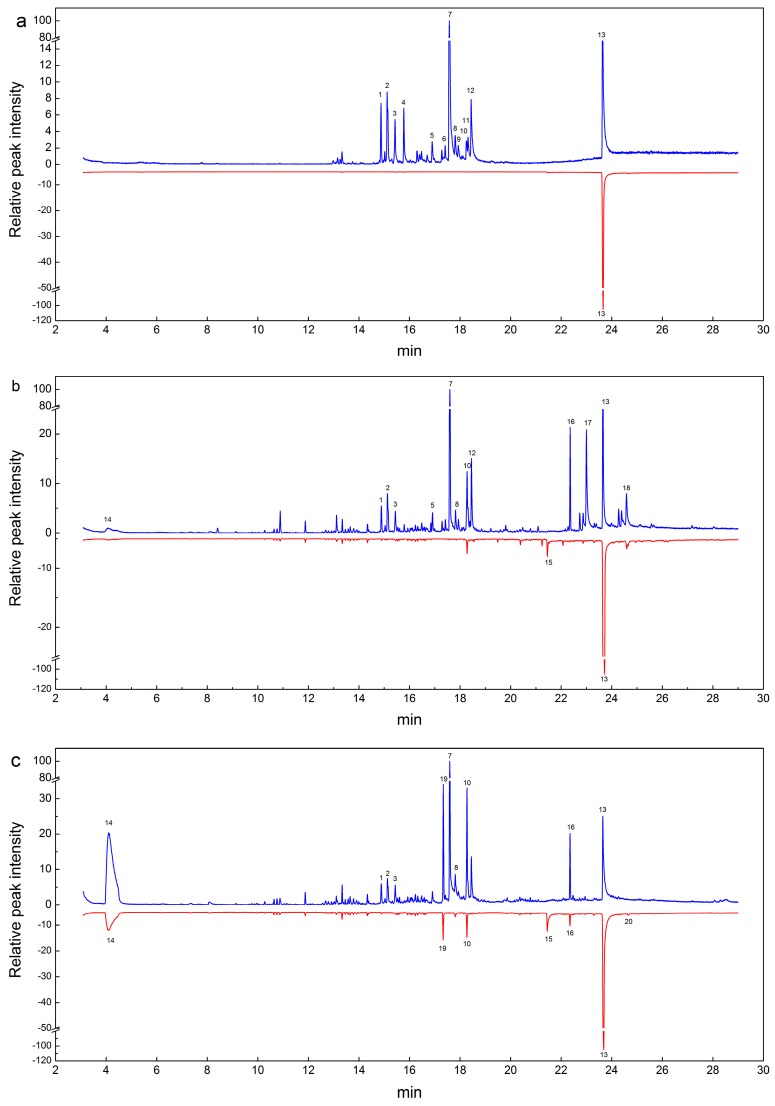
Respective total ion chromatograms (TICS) of *Pterocarpus santalinus* (blue) and *P. tinctorius* (red): (**a**) ethanol and water (EW) extracts, (**b**) ethyl acetate (EA) extract*s*, and (**c**) benzene–alcohol (BE) extracts.

Extraction of *P. santalinus* and *P. tinctorius* samples was conducted under three different solvent systems: EW, EA, and BE, respectively. The extracts were then analyzed by GC-MS. In the subsequent GC-MS analyses, one sample in each species was used as a representative. The typical GC-MS total ion chromatograms (TICS) of the heartwood extracts of *P. santalinus* and *P. tinctorius* are shown in Figure 1. As shown in Figure 1, it is obvious that significant differences appear in the heartwood extracts of *P. santalinus* and *P. tinctorius.* A greater number of peaks are observed in the TICS curves of the heartwood extracts of *P. santalinus* than in those of *P. tinctorius.* TICS curves of *P. tinctorius* of three different kinds of extracts are similar (Figure 1). On the contrary, TICS curves of three different kinds of extracts of *P. santalinus* are significantly different.

For three different kinds of extraction solvents, the distinctions were a consequence not only of differences in the number of detected molecules, but also in the relative content of peaks differed between *P. santalinus* and *P. tinctorius.* The peak area was the analytical signal for the relative content of peaks. The relative content was calculated by area normalization and the average value of the three replicates. Peaks whose area was above 1% were tentatively identified by matching their mass spectra with those in the NIST 11 library and in the literature, as summarized in Table 1. A total of twenty volatile compounds were detected and tentatively identified in three kinds of extracts, and these compounds included alcohols, stilbenoids, esters, aromatic hydrocarbons, ketones, miscellaneous, phenols, and flavonoids.

In the case of the EW extract, twelve distinct compounds were only observed in the samples of *P. santalinus*, and the principal compounds included spathulenol (peak 7, 46.89%), beta-Eudesmol (peak 2, 6.12%), 2,3,3-Trimethyl-2-(3-methylbuta-1,3-dienyl)-6-methylenecyclohexanone (peak 12, 5.77%) and 6-Isopropenyl-4,8a-dimethyl-1,2,3,5,6,7,8,8a-octahydro-naphthalen-2-ol (peak 4, 4.16%). It was noted that spathulenol showed the largest relative content in the *P. santalinus,* while it was not detected in the *P. tinctorius*. Thus, spathulenol may play a key role in wood identification between these two species. A peak at 23.65 min was the common peak between these two species. This peak has a molecular weight of 256, corresponding to pterostilbene. Pterostilbene, with a molecular formula of C_16_H_16_O_3_, is a stilbene compound isolated from *Pterocarpus* species [27]. The relative content of pterostilbene was significantly different between *P. santalinus* (16.51%) and *P. tinctorius* (99.2%), which indicated that pterostilbene may also be the critical compound for the wood classification.

For the EA extract, a total of fourteen compounds were detected in the two *Pterocarpus* species. There are two common compounds between the two species, including dibutyl phthalate (peak 10) and pterostilbene (peak 13). Dibutyl phthalate presents a higher relative content in *P. santalinus* (5.52%) compared to *P. tinctorius* (1.25%). Whereas, a higher amount of pterostilbene was observed in the *P. tinctorius* (97.16%) than in the *P. santalinus* (13.87%). 4-*t*-Butyl-2-[4-nitrophenyl]phenol (peak 15, 1.59%) was specific to the *P. tinctorius.* In addition, the remaining eleven compounds were exclusively detected in the *P. santalinus*. Among them, spathulenol (peak 7, 28.14%), 2,3,3-Trimethyl-2-(3-methylbuta-1,3-dienyl)-6-methylenecyclohexanone (peak 12, 5.46%), 2,2’-Methylenebis(6-tert-butyl-4-methylphenol) (peak 16, 5.04%), hexadecanoic acid, and 2-hydroxy-1-(hydroxymethyl)ethyl ester (peak 17, 9.16%) were the main compounds.

Towards the BE extract, it should be noted that a big peak at 4.1 min assigned for p-Xylene was detected in the BE extract. This compound was from the benzene–ethyl alcohol solvent, which may have a bad impact on the wood identification. Besides, six compounds were found both in the two species, of which, diisobutyl phthalate was one compound only detected in the BE extract. 4H-1-Benzopyran-4-one,5-hydroxy-2-(4-hydroxyphenyl)-7-methoxy- (peak 20, 1.16%) was specific to *P. tinctorius*. The following five distinct compounds were also found in the GC-MS analyses of *P. santalinus*; 2-naphthalenemethanol,1,2,3,4,4a,5,6,7-octahydro-a,a,4a,8-tetramethyl-, (2R,4aR)- (peak 1, 1.33%), beta-eudesmol (peak 2, 2.26%), α-bisabolol (peak 3, 1.33%), spathulenol (peak 7, 18.44%), and 2,3,3-trimethyl-2-(3-methylbuta-1,3-dienyl)-6-methylenecyclohexanone (peak 12, 2.77%).

### 2.2. Multivariate Analyses

**Figure 2:**
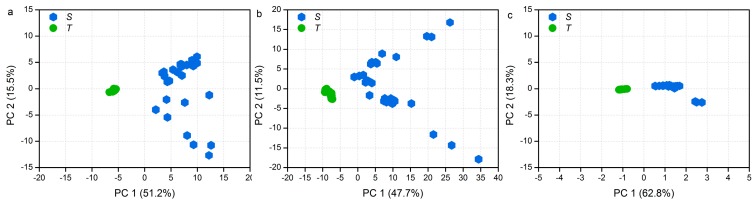
Principal component analysis (PCA) score plot: (**a**) EW extract, (**b**) EA extract, and (**c**) BE extract. (S) *P. santalinus* and (T) *P. tinctorius*.

**Figure 3:**
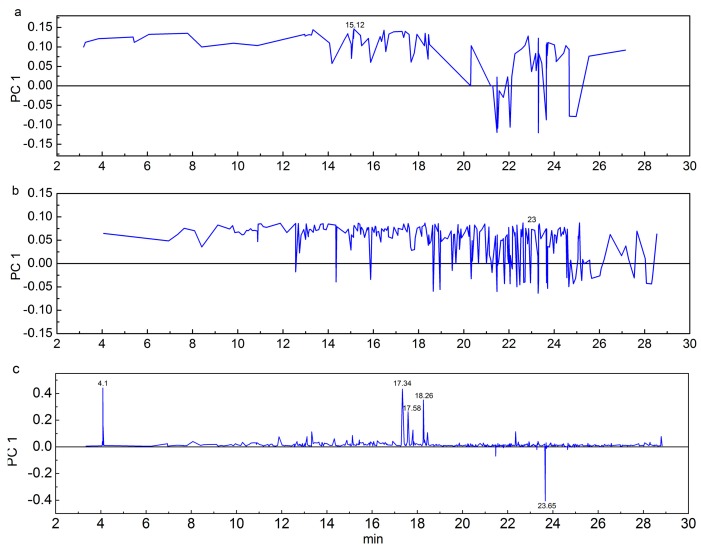
Loading plot of PC1: (**a**) EW extract, (**b**) EA extract, and (**c**) BE extract.

PCA methods were applied to the processed dataset to visualize the clustering trends between the two *Pterocarpus* species. Samples with similar values for the variables explained by the principal components appeared close together in the PCA score plot [28]. For the EW extract, the first principal component (PC1) represented 51.2% of the variance and the second principal components represented 15.5% of the total variance. Along the PC1 axis, the left side of the plot shows the cluster of *P. tinctorius* samples, and the right side depicts the *P. santalinus* samples (Figure 2a). Similar results were also observed both in the EA extract and the BE extract (Figure 2b,c). The existing distinguish was thought to be derived from the difference of wood species. The loading plot can further illustrate the key variance responsible for the distinction between the groups, and the loading plot of PC1 shows in the Figure 3. Peak at 15.12 min in the EW extract and peak at 23 min in the EA extract present high contribution for the classification (Figure 3a,b), while differences of the relative content of these peaks still exist in the *P. santalinus* samples (Figure S1). This may be the reason why samples from *P. santalinus* were spread in the PCA score plot of the EW extract and the EA extract. Furthermore, the samples cultivated in different regions with different growth conditions could also affect the result of score plot. Additionally, the BE extract seems to provide the best separation because of the tightest sample distribution on the score plot. As shown in the loading plot of PC1 of the BE extract, *p*-Xylene (4.1 min) derived from the benzene–ethyl alcohol solvent also presents high contribution for the classification except for the peaks at 17.34, 17.58, 18.26, and 23.65 min (Figure 3c). This phenomenon is detrimental to wood identification in practice because the difference from the chemical composition of wood would be weakened.

OPLS-DA, a supervised multivariate analysis method, was constructed to further understand the differences between *P. santalinus* and *P. tinctorius* and to provide the information of the correlations between specific markers and each particular wood species [29,30]. The objective of OPLS-DA is to separate the systematic variation in X into two parts, one part which is linearly related to Y, and another part is orthogonal to Y, which leads to better class resolution in a discriminant problem [31]. Classification models were established using all the samples from the training set (see Table 2). The supervised OPLS-DA models for samples subjected to different kinds of extraction solvents all exhibited accurate differentiation performance of the explained fraction of variance of classes (R^2^Y = 0.949–0.978) and the cross-validated fraction of variance of classes (Q^2^ = 0.944–0.97) according to cross-validation, which showed acceptable predictability for the wood species. To further validate the models, all the samples from the test set were used to test their predictive quality. The models generated with the GC-MS data of the EW extract present the highest predictive capacity (100%) for samples from the test set. As for the models based on the data of the EA extract and BE extract, one sample from the test set was classified incorrectly, and the predictive accuracy was only 83.33%. Due to the advantages of low toxicity, easy availability, low-cost, and highest predictive accuracy, EW was considered as a more suitable solvent in the wood identification of *P. santalinus* and *P. tinctorius* using GC-MS.

**Figure 4:**
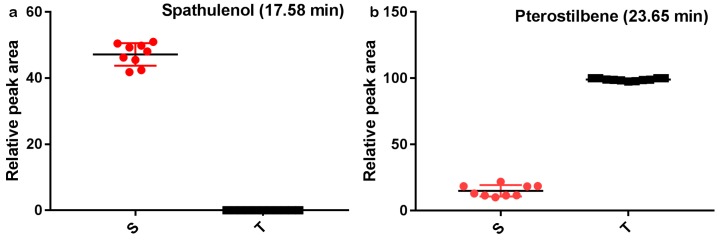
Quantification of marker compounds selected by variable importance in projection (VIP) > 3 and *p* < 0.05 in the EW extract of *P. santalinus* (S) and *P. tinctorius* (T) using GC-MS: (**a**) Spathulenol (17.58 min) and (**b**) Pterostilbene (23.65 min).

**Figure 5:**
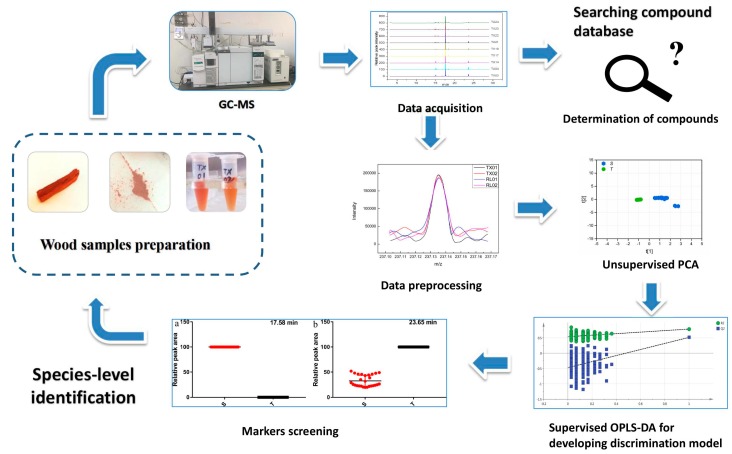
A protocol of GC-MS and multivariate analyses for the wood identification: Principal component analysis (PCA) and (orthogonal partial least square-discriminant analysis OPLS-DA).

Variable importance in projection (VIP) analysis was employed to provide the order of contribution of variables to the separation of clustering [29]. The contribution of the variables between the two groups increased with increasing VIP value [31]. The variables whose VIP value was higher than 3 and *p*-value obtained from the *t*-test was lower than 0.05, were selected as potential marker compounds with the significant differences between *P. santalinus* and *P. tinctorius*. Due to the highest predictive accuracy, only the EW extract was performed for the selection of potential marker compounds. For the EW extract, peaks at 17.58 (VIP value is 6.49) and 23.65 min (VIP value is 3.96) were considered as potential marker peaks. It indicated that spathulenol and pterostilbene were the marker compounds for the wood discrimination between *P. santalinus* and *P. tinctorius*, which was consistent with the previous analysis results (Figure 4). The results suggested that GC-MS coupled with statistical analyses had a high development and application potential to the wood trade and technology. A protocol suitable for wood identification using GC-MS and multivariate analyses was developed in this study (Figure 5).

## 3. Materials and Methods

### 3.1. Materials and Chemicals 

Twelve of the analyzed *P. santalinus* heartwood specimens and fourteen of *P. tinctorius* heartwood specimens were collected from curated xylaria collections (Table 3). All the specimens contain the information of their botanical voucher ID or scientific validation. Among these specimens, nine specimens of *P. santalinus* and eleven specimens of *P. tinctorius* were selected randomly as the training set for creating the classification models. The remaining six specimens were used as the test set for validation purposes. Ethanol absolute, ethanol (95%), and benzene were purchased from Beijing Chemical Works (Beijing, China). Ethyl acetate was bought from Fuchen Chemical Reagent Company (Tianjin, China). 

### 3.2. Sample Preparation

To overcome the shortcomings of the traditional wood identification methods and develop a practical method as alternative, a mild condition for extraction was used in this study. All heartwood specimens were dried at room temperature and ground into a fine powder using a 6770 Freezer/Mill (Spex SamplePrep, Metuchen, NJ, USA) with cycle conditions consisting of a 1 min precool, 2 min crush, and 1 min cool. Approximately 5 mg of heartwood powder was extracted ultrasonically with 1 mL solvent for 30 min at 25 °C (ultrasonic power of 50 W). The mixture was then centrifuged at 750× *g* for 2 min. Finally, ~1 μL supernatant of each sample was used for GC-MS analysis. Three different kinds of solvents were used in this study, including 1:1 (*v*/*v*) mixture of ethanol and water (EW), ethyl acetate (EA), and benzene–ethanol (BE). 

### 3.3. Apparatus and Chromatographic Conditions

GC-MS analyses were performed on a GC-MS (Agilent 7890A, Santa Clara, CA, USA) equipped with a 5975C mass spectrometer (Avondale, PA, USA). A HP-5MS capillary fused silica column (30 m × 250 μm i.d., 0.25 μm film thickness) was used for separation, and helium (99.999%) was used as carrier gas with a flow rate of 1 mL/min. The oven temperature program initiated at 60 °C, held for 2 min, then increased at 10 °C/min to 280 °C, and then held at this temperature for 5 min. The injector temperature was 260 °C. A sample of 0.5 μL was injected in the split mode injection. The mass spectrometric data were recorded in the range of 50 to 500 *m*/*z*. Three replicates were analyzed per sample.

### 3.4. Determination of Chemical Compounds

Peak deconvolution is a critical stage to discriminate coeluting compounds from multiple ions. Automated mass spectral deconvolution and identification system (AMDIS) is a common method for deconvolution of GC-MS data. Thus, the components eluting from GC-MS were extracted in the AMDIS and then mass spectral fragmentation patterns were compared with those stored in the National Institute of Standards and Technology (NIST, Gaithersburg, MD, USA) libraries and the mass spectra reported from the literatures.

### 3.5. Multivariate Analyses

All the GC-MS raw files were converted into NETCDF format, and then peak detection, identification, and alignment were performed using MS-DIAL software (v 2.74) [32]. Aligned peak area data based on the full GC-MS spectra were exported and normalized for the subsequent multivariate statistical analysis.

For EW extract and EA extract, a total of 78 GC-MS files were used for the subsequent statistical analysis (60 files as the training set and 18 files as the test set). For BE extract, 75 GC-MS files were used for the subsequent statistical analysis (57 files as the training set and 18 files as the test set) because three files from one sample of *P. santalinus* are invalid.

Principal component analysis (PCA) and OPLS-DA were widely applied with unsupervised and supervised test methods. These methods can reduce the dimensionality of raw data and provide a visualizing result for easy interpretation of complicated raw data. PCA and OPLS-DA analyses were conducted by SIMCA-P (14.1 Umetrics, Umea, Sweden) software. SPSS 22.0 (SPSS, Chicago, IL, USA) was used for the student’s *t* test to determine if the data of the two species are significantly different.

## 4. Conclusions

A GC-MS and multivariate analyses approach was developed to establish a protocol for the discrimination of the endangered *P. santalinus* and non-endangered *P. tinctorius* wood species, which could be potentially used for wider application in wood identification field. A total of twenty volatile compounds were detected and tentatively identified in the three kinds of extracts, and these compounds included alcohols, stilbenoids, esters, aromatic hydrocarbons, ketones, miscellaneous, phenols, and flavonoids. Both the number of detected compounds and their relative content significantly differed between *P. santalinus* and *P. tinctorius.* Compared to the ethyl acetate extract and benzene–ethanol extract, the 1:1 mixture of ethanol and water extract performed with high predictive accuracy (100%). Spathulenol (17.58 min) and pterostilbene (23.65 min) were considered as the potential markers to characterize and differentiate 1:1 mixture of ethanol and water extracts of these two species. The results suggested that GC-MS was an effective analytical method for wood identification at the species level.

In the further study, a large-sized sample and more extraction methods, including soxhlet, would be inspected to investigate the effect of sample size and extraction methods on the classification results.

## Figures and Tables

**Table 1:** Chemical composition of wood extract analyzed by gas chromatography–mass spectrometry (GC-MS).

ID	RT (min)	Molecular Formula	Possible Compounds	Classification	Relative Content for *P. santalinus* (%) ^1^			Relative Content for *P. tinctorius* (%) ^1^		
					EW ^2^	EA ^2^	BE ^2^	EW ^2^	EA ^2^	BE ^2^
1	14.87	C_15_H_26_O	2-Naphthalenemethanol,1,2,3,4,4a,5,6,7-octahydro-a,a,4a,8-tetramethyl-, (2*R*,4a*R*)-	Alcohol	3.37	1.68	1.33	- ^3^	-	-
2	15.12	C_15_H_26_O	beta-Eudesmol	Alcohol	6.12 (0.45)	3.41	2.26	-	-	-
3	15.43	C_15_H_26_O	α-Bisabolol	Alcohol	3.51	1.64	1.33	-	-	-
4	15.78	C_15_H_24_O	6-Isopropenyl-4,8a-dimethyl-1,2,3,5,6,7,8,8a-octahydro-naphthalen-2-ol	Alcohol	4.16 (0.38)	-	-	-	-	-
5	16.9	C_15_H_26_O	a-Eudesmol	Alcohol	1.59	1.1	-	-	-	-
6	17.41	C_15_H_24_O	2-(4a,8-Dimethyl-1,2,3,4,4a,5,6,7-octahydro-naphthalen-2-yl)-prop-2-en-1-ol	Alcohol	1.32 (0.40)	-	-	-	-	-
7	17.58	C_15_H_24_O	Spathulenol	Alcohol	46.89 (0.98)	28.14 (0.32)	18.44	-	-	-
8	17.81	C_15_H_24_O	2-(4a,8-Dimethyl-1,2,3,4,4a,5,6,7-octahydro-naphthalen-2-yl)-prop-2-en-1-ol	Alcohol	2.03 (0.35)	1.26	1.33 (0.80)	-	-	0.74
9	17.93	C_15_H_24_O_2_	Murolan-3,9(11)-diene-10-peroxy	Miscellaneous	1.14	-	-	-	-	-
10	18.26	C_16_H_22_O_4_	Dibutyl phthalate	Ester	1.25	5.52	6.86 (0.35)	-	1.25	3.3
11	18.32	C_15_H_22_O	Longipinocarvone	Ketone	1.70 (0.46)	-	-	-	-	-
12	18.43	C_15_H_22_O	2,3,3-Trimethyl-2-(3-methylbuta-1,3-dienyl)-6-methylenecyclohexanone	Ketone	5.77 (0.51)	5.46	2.77 (0.31)	-	-	-
13	23.65	C_16_H_16_O_3_	Pterostilbene	Stilbenoid	16.51 (1.6)	13.87	8.56 (0.51)	99.2 (1.1)	97.16	64.12 (0.65)
14	4.1	C_8_H_10_	*p*-Xylene	Aromatic Hydrocarbons	-	2.05 (0.66)	41.18 (0.97)	-	-	22.79 (0.47)
15	21.45	C_16_H_17_NO_3_	4-*t*-Butyl-2-[4-nitrophenyl]phenol	Miscellaneous	-	-	-	-	1.59	3.06
16	22.35	C_23_H_32_O_2_	2,2’-Methylenebis(6-tert-butyl-4-methylphenol)	Phenol	-	5.04	3.26	-	-	1.62
17	23	C_19_H_38_O_4_	Hexadecanoic acid, 2-hydroxy-1-(hydroxymethyl)ethyl ester	Miscellaneous	-	9.16 (0.42)	-	-	-	-
18	24.58	C_21_H_42_O_4_	Octadecanoic acid, 2,3-dihydroxypropyl ester	Miscellaneous	-	3.15 (0.32)	-	-	-	-
19	17.34	C_16_H_22_O_4_	Diisobutyl phthalate	Ester	-	-	5.52	-	-	3.22
20	24.64	C_16_H_12_O_5_	4H-1-Benzopyran-4-one, 5-hydroxy-2-(4-hydroxyphenyl)-7-methoxy-	Flavonoid	-	-	-	-	-	1.16

^1^ The percentage was calculated based on the peak area. The values in the parentheses are the deviations of three replicates. Deviations lower than 0.3% are not listed in the Table. ^2^ EW, ethyl alcohol extract; EA, ethyl acetate extract; BE, benzene–ethyl alcohol extract. ^3^ -: not detected.

**Table 2:** Classification capacity of three models based on the orthogonal partial least square-discriminant analysis (OPLS-DA).

Models	R^2^X	R^2^Y	Q^2^	Accuracy (%)
EW	0.902	0.949	0.944	100.00
EA	0.893	0.978	0.963	83.33
BE	0.743	0.970	0.97	83.33

EW, 1:1 mixture of water and ethyl alcohol extract; EA, ethyl acetate extract; BE, benzene–ethyl alcohol extract.

**Table 3:** Wood samples examined.

Species	Sample ID	Voucher ID	Origin	N
*Pterocarpus santalinus* L.f.	TX01	Verified by DNA [6]	India	1
	TX03	Verified by DNA [6]	India	1
	TX04	Verified by DNA [6]	India	1
	TX14	Roy. Bot. Gard.	India	1
	TX17-TX24	LB-03494	India	8
*Pterocarpus tinctorius* Welw.	W006	XD-01698	Congo	1
	W008-3-W008-12	XCY-00326	Congo	10
	W37619	BR, LUA, LISC, MAD	Angola	1
	W37621	BR, LUA, LISC, MAD	Angola	1
	W37622	BR, LUA, LISC, MAD	Angola	1

N, numbers of per samples. Voucher ID, botanical voucher ID of samples.

## Supplementary Materials

The supplementary materials are available online.
