# Supplementary figures and images for: A GC-MS Protocol for Separating Endangered and Non-endangered Pterocarpus Wood Species
Source: Molecules. 2019 Feb 22;24(4):799. doi: 10.3390/molecules24040799 (PMC6413215; doi:10.3390/molecules24040799)

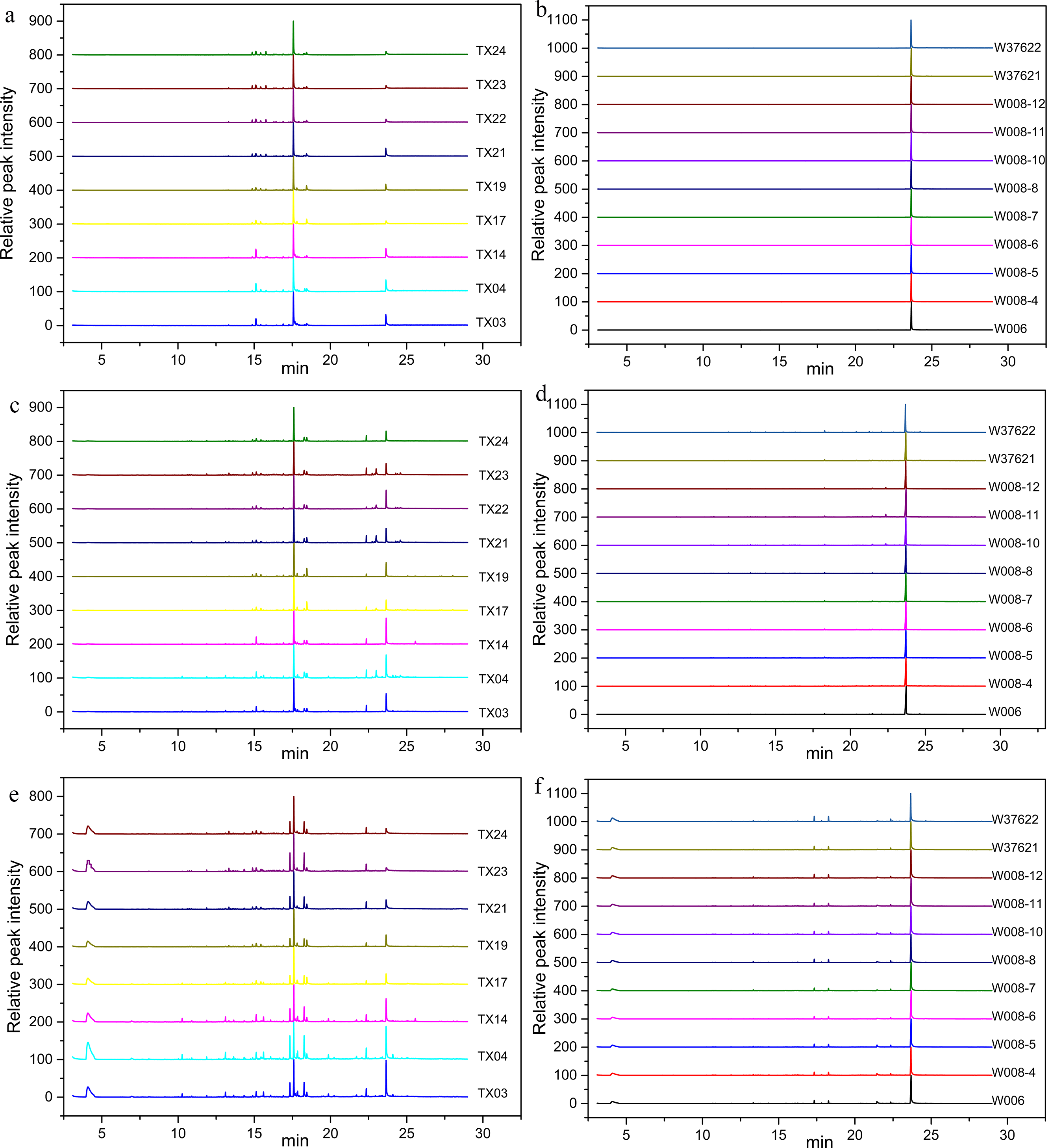

Supplement: Supplementary file 1 [file molecules-24-00799-s001.zip › Supplementary files/Figure S1.tif]
