# Supplementary material for: A GC-MS Protocol for Separating Endangered and Non-endangered Pterocarpus Wood Species
Source: Molecules. 2019 Feb 22;24(4):799. doi: 10.3390/molecules24040799 (PMC6413215; doi:10.3390/molecules24040799)
Supplement: Supplementary file 1 [file molecules-24-00799-s001.zip › Supplementary files/Figure S1.doc]

**Figure S1.** The total ion chromatograms (TICS) of heartwood extracts of samples from the training set: (a) EW extract of *P. santalinus*, (b) EW extract of *P. tinctorius*, (c) EA extract of *P. santalinus*, (d) EA extract of *P. tinctorius*, (e) BE extract of *P. santalinus*, (f) BE extract of *P. tinctorius*.
